# Dr. James E. Reeves

**Published:** 1896-03

**Authors:** 


Dr. James E. Reeves died at Chattanooga, Tenn., January 4, 1896, of what was supposed to be cancer of the liver. Dr. Reeves was born in Rappahannock county, Va., 1829. He graduated from the University of Pennsylvania in 1860. He lived for some years in Wheeling, W. Va., and then removed to Chattanooga, where he resided until his death.

Dr. Reeves, says the *Southern Medical Record*, was one of the founders of the American Public Health Association and was its president in 1885. His works in microscopy and hygiene are well known. His contributions to the medical press were numerous and covered a wide range of subjects. About a year ago he published a small work on the use of the microscope. Dr. Reeves was a gentleman of the old régime, and his death will be a grievous loss to many friends in and out of the profession.

